# Economic Circumstances in Childhood and Subsequent Substance Use in Adolescence – A Latent Class Analysis: The youth@hordaland Study

**DOI:** 10.3389/fpsyg.2019.01115

**Published:** 2019-05-14

**Authors:** Jens Christoffer Skogen, Børge Sivertsen, Mari Hysing, Ove Heradstveit, Tormod Bøe

**Affiliations:** ^1^Centre for Alcohol and Drug Research, Stavanger University Hospital, Stavanger, Norway; ^2^Department of Health Promotion, Norwegian Institute of Public Health, Bergen, Norway; ^3^Department of Research and Innovation, Helse Fonna HF, Haugesund, Norway; ^4^Department of Mental Health, Norwegian University of Science and Technology, Trondheim, Norway; ^5^Regional Centre for Child and Youth Mental Health and Child Welfare, NORCE Norwegian Research Centre, Bergen, Norway; ^6^Department of Psychosocial Science, Faculty of Psychology, University of Bergen, Bergen, Norway

**Keywords:** economic circumstances, economic volatility, adolescence, substance use, alcohol use, relative poverty

## Abstract

The aim of the present study was to investigate the association between longitudinal registry-based data on family income during childhood and self-reported substance use in adolescence, including potential alcohol- or drug problems. Data from the Norwegian population-based youth@hordaland-survey was employed, and the analyzed included *n* = 8,983 adolescents aged 16–19 years. This information was linked to registry-based information about childhood family income for seven consecutive years prior to adolescents’ participation in the youth@hordaland-survey. Latent class analyses (LCA) were used to examine associations between patterns of family economic circumstances in childhood and subsequent substance use in adolescence. Based on the LCA, we identified four distinct patterns of family economic circumstances: a ‘never poor’ (89.3%) group, followed by two groups characterized by moving in (3.0%) or out (4.6%) of poverty, and a final ‘chronically poor’ (3.1%) group. Several findings were of interest: the chronically poor reported less daily snus use, fewer had tried alcohol, were less likely to report frequent intoxication, and less prone to have potential alcohol- or drug-related problems compared to all other groups. They were also less likely to have tried any illicit drug compared to those moving in or out of poverty. Finally, the chronically poor reported more daily smoking than the never poor group, but less daily smoking than the moving out of poverty group. The never poor group was less likely to have tried any illicit drugs compared to the groups moving into or out of poverty, and less likely to smoke daily compared to the moving out of poverty group. In other words, the present study somewhat surprisingly suggested lower substance use among the chronically poor adolescents compared to other adolescents on several of the measures of substance use.

## Introduction

Adolescence is an important transitional period for future health – for both the individual, and generations to come (Sawyer et al., 2012; Patton et al., 2016), and also a period where many health-related behaviors are established (World Health Organization [WHO], 2018). Debut of alcohol and other substance use predominantly occur during this developmental period (Zucker, 2008; Skogen et al., 2016).

Socioeconomic status (SES) is one of several factors that influence substance use among individuals across the lifespan (Jones et al., 2016). Numerous studies have investigated the association between different SES indicators and the use of alcohol and other substances during adolescence and young adulthood (Humensky, 2010; Patrick et al., 2012; Finch et al., 2013; Kendler et al., 2014; Latvala et al., 2014; Lui et al., 2015; Pedersen et al., 2015; Charitonidi et al., 2016; Non et al., 2016; Gomes de Matos et al., 2017; Jang et al., 2017; Pape et al., 2017, 2018; Lee et al., 2018). The nature of this association is complex, and the link seems to be dependent on both how SES is measured and operationalized, the substance in question, the type of use, and the participating adolescents’ age (Ensminger et al., 2000; Boyce et al., 2006; Currie et al., 2008; Kendler et al., 2014; Charitonidi et al., 2016; Pape et al., 2018). For example, Kendler and colleagues found that higher SES, as indicated by family income, parental education, and occupational status, predicted increased alcohol consumption in early adolescence and heavy episodic drinking in late adolescence (Kendler et al., 2014). In contrast, lower SES predicted alcohol-related behavioral problems, as well as high-quantity alcohol consumption later on (Kendler et al., 2014). Furthermore, Charitonidi and colleagues reported that there might be a differential association across SES indicators (such as parental education and perceived family income) and substance use, as well as different association between various indicators of SES and various substances (Charitonidi et al., 2016). Two recent publications by Pape and colleagues, however, first found that both high level alcohol consumption and drunkenness were more prevalent in lower social strata (as measured by parental education) (Pape et al., 2017). In their second publication, they found that associations between low SES and different alcohol-behavior outcomes varied, where the strongest association was found for deviant and harmful drinking compared to alcohol use *per se* (Pape et al., 2018).

With regards to the association between individual-level SES and substance use, all of the previously published studies are, to the best of our knowledge, based on self-reported or parental-reported SES (Humensky, 2010; Patrick et al., 2012; Finch et al., 2013; Kendler et al., 2014; Latvala et al., 2014; Lui et al., 2015; Charitonidi et al., 2016; Non et al., 2016; Gomes de Matos et al., 2017; Jang et al., 2017; Pape et al., 2017, 2018; Lee et al., 2018). The use of self-reported SES may be prone to bias and systematic missing data (Ensminger et al., 2000; Svedberg et al., 2016).

Furthermore, most previous studies only measure SES once (Finch et al., 2013; Charitonidi et al., 2016; Collins, 2016; Gomes de Matos et al., 2017; Pape et al., 2017, 2018; Lee et al., 2018). In contrast, few studies have examined how changes in financial circumstances affect adolescent alcohol and substance use. While some families may be poor during all of childhood and adolescence, other families’ financial status may change over this period of time. This aspect is important and may provide a more dynamic and relevant representation of the influence of SES on alcohol/substance use. One exception is a study that found that moving into low family income was linked to increased alcohol use in adolescence, whereas neither the group with increasing family income nor the group with stable low income were associated with adolescents’ alcohol use (Poonawalla et al., 2014). A few other studies have also measured SES at multiple time-points (Humensky, 2010; Patrick et al., 2012; Kendler et al., 2014; Latvala et al., 2014; Lui et al., 2015; Non et al., 2016); however, all these studies were dependent on informant-reported SES.

The present study investigates the association between longitudinal registry-based data on family income during childhood and self-reported substance use in adolescence, including potential alcohol- or drug problems. We aim to further develop the knowledge-base by the (i) use a longitudinal objective measure of family income and (ii) the use of several self-reported substance-related behaviors.

## Materials and Methods

### Study Population

Data from the Norwegian population-based youth@hordaland-survey was employed, and included 9,154 adolescents aged 16–19 years. Data collection was carried out during the spring of 2012. The main aim of youth@hordaland-survey was to assess mental health problems among all upper secondary school-aged adolescents in Hordaland County. All adolescents within the relevant age range received an e-mail with information about the study. One classroom school hour was allotted for the adolescents to complete the questionnaire. The e-mail contained information about the study, a link to the survey, and a username and password used for logging into the survey. Information was sent by post to those not in school. The questionnaire was web-based and included several different areas such as mental health issues, lifestyle behaviors, and use of healthcare and social services. Also, a request for permission to obtain official school data, and to link the information with national registries was included in the questionnaire. Participants were not compensated for their effort, but all adolescents that were eligible for participation entered into a lottery where they could prizes ranging from an iPad to movie tickets. Those in school not wishing to participate or did not consent to participation were allowed to use the allotted school hour for alternative school-related tasks. Uni Research Health collaborated with Hordaland county Council to conduct the study.

### Study Setting and Overall Characteristics of Study Population

The Hordaland County population is in general considered to be representative of Norway when it comes to sociodemographic composition. The median household income is also comparable to the national average (Statistics Norway, 2013). During 2005–2010, the average proportion of children characterized as relatively poor (see details below) in Hordaland county was slightly lower (7.3%) than for Norway as a country (8.9%). Official data indicate that 92% of all adolescents in Norway aged 16–18 years attended high school compared with 98% in the current sample at the year of inclusion (Statistics Norway, 2018). The grade point average (GPA) in the current sample was somewhat lower than the GPA in Hordaland county, but similar to the national GPA (Hysing et al., 2016).

### Demographics of Study Population

Gender and date of birth were retrieved from the Norwegian National Population Registry. Age was estimated using information about date of birth and date of study participation. The following demographic information was also reported by the participating adolescents (Skogen et al., 2018):

• Ethnicity: Norwegian (96.3%) or foreign;
• Family structure: Single-parent (15.0%) or two-parent households;
• Parental educational attainment: Elementary (3.7%), intermediate (30.7%), and higher;
• Parental work affiliation: Work (93.4%)], benefits (3.8%) or other (including students, retirees, and stay-at-home).

### Indicator of SES: Family Income

Information about income is based on tax return data from the Norwegian Tax Administration made available by the Norwegian national income registry. The youth@hordaland-population was linked to the national registry, and information about family income seven consecutive years prior to participation in the youth@hordaland-survey was extracted. The information about income is used by the Norwegian Government to estimate taxation, and is therefore deemed to be of high quality – reliable and precise. By the use of personal identification numbers, we could retrieve the disposable equivalized household income for the period from 2004 when the participants were aged 8–11, until 2010 (aged 14–17). Equivalized household income is a measure of household income which is adjusted in relation to an equivalence scale that enables comparison between households of different size and composition. Conceptually, it can be understood as an indicator of the economic resources that are available to a standardized household, while accounting for inflation/changes in median income over time. The equivalence scale employed in the present study is the scale put forth by the European Union (a modified version of the OECD equivalence scale) where the first adult is given a weight of 1, subsequent adults are given a weight of 0.5 and each child <14 years of age is given the weight 0.3 (Hagenaars et al., 1994; Vos and Zaidi, 1997). By the use of this measure of family income, we estimated the proportion of adolescents in relative poverty, defined as having an equivalized household income below 60% of the equivalized national median income for each year (e.g., we used the median income for 2010 to calculate relative poverty proportions for 2010). This definition is in correspondence to the one employed in income inequality statistics by the European Union (Bardone and Gulo, 2005; Eurostat, 2016).

### Self-Reported Outcomes: Substance Use

The adolescents responded to questions regarding having tried alcohol or illicit drugs, daily smoking, and daily snus use (a form of smokeless tobacco). Also, the amount of alcohol usually consumed and potential alcohol- or drug problems were reported.

#### Use of Tobacco Products

Participants were asked whether they had tried smoking cigarettes or snus [a form of smokeless tobacco traditionally used in Norway and Sweden (Øverland et al., 2013)]. Those who reported having tried smoking cigarettes were prompted to indicate if they smoked, and whether they smoked on a daily basis. The same prompt was repeated for those having tried snus. Two binary variables were constructed, coded ‘1’ for those reporting daily smoking/snus use and ‘0’ for the remaining participants.

#### Ever Tried Alcohol

A dichotomous variable based on the question ‘Have you ever tried alcohol?’ (Yes/No).

#### Ever Tried Illicit Drugs

A dichotomous variable based on the question ‘Have you ever tried hash, marijuana or other narcotic substances?’ (Yes/No).

#### Usual Alcohol Consumption

Five questions about self-reported glasses of beer, cider, wine, spirits and illegally distilled spirits usually consumed during a 14-day period were added together. Each of the questions were worded: “How many glasses of X do you usually consume during a 14-day period?”

#### Frequent Alcohol Intoxication

Frequency of alcohol intoxication was assessed using the question: ‘Have you ever consumed so much alcohol that you were clearly intoxicated (drunk)?’ Five response categories ranging from ‘No, never’ to ‘Yes, more than 10 times’ were available. Frequent alcohol intoxication was defined as answering ‘Yes, more than 10 times’ (Skogen et al., 2014), and a dichotomous variable was created.

#### Alcohol and Drug-Related Problems

An indicator for potential alcohol and drug-related problems was constructed using the six-item, validated scale CRAFFT (acronym for the keywords of each question: Car, Relax, Alone, Forget, Friends, Trouble) were administered to all participants. CRAFFT was developed to screen adolescents for high-risk alcohol and other drug use disorders simultaneously, and does not differentiate between types of substances, as each question is worded in relation to “alcohol or drugs” combined. CRAFFT does not include any items related to consumption levels or frequency of consumption. The summed CRAFFT-score range from 0 to 6, where a higher score indicate more alcohol and drug-related problems. This scale was designed to identify possible alcohol-and drug related problems among adolescents, and has been demonstrated to have acceptable sensitivity and specificity at a cut-off of equal to or more than 2 (Dhalla et al., 2011), and it has been found to have a good concurrent validity in youth@hordaland-sample (Skogen et al., 2013). In the present study, a dichotomous variable separating those above the cut-off of ≥2 on CRAFFT from those below the cut-off was constructed.

### Statistical Analyses and Analytical Model

The statistical analyses are mainly similar to previous publications using the same data using latent class analysis (LCA) (Bøe et al., 2017; Sivertsen et al., 2017). Being a person-centered approach, LCA was used to identify groups of participants who showed a similar pattern of family income across follow-up (2004–2010). Akaike information criterion (AIC), Bayesian information criterion (BIC) and sample-size adjusted BIC (adj BIC) was used to inform our decision about the number of classes to retain. Additionally, we employed relative entropy to ascertain the quality of classification. Finally, we used the Vuong-Lo-Mendell-Rubin (VLMR) adjusted likelihood ratio test to test whether a model with one less class performs just as well. Using an iterative approach, we started with one class, and increased the number of classes until the abovementioned fit criteria indicated an adequate model. Model characteristics, statistic criteria, parsimony and meaningfulness of the classes was considered collectively when deciding which model to retain. Mplus V.7.4 was used for the LCA (Muthén and Muthén, 2012). For further information and details about the family income classes identified in youth@hordaland (see Bøe et al., 2017; Sivertsen et al., 2017). As a previous study employing the youth@hordaland sample have shown that there are important differences in the use of alcohol, tobacco, and illicit drugs between ethnic groups (Skogen et al., 2018) and differential proportion of ethnic minorities in previously identified classes (Bøe et al., 2017), we included ethnicity in our analytical model. Using Mplus, the proportion and 95% confidence intervals for each outcome was computed and compared across the identified classes.

### Ethics Statement

The questionnaires used in the youth@hordaland study were web-based, and electronic informed consent was obtained from all participants. The study was approved by The Regional Committee for Medical and Health Research Ethics in Western Norway as appointed by the Norwegian Ministry of Education and Research^1^. Adolescents aged 16 years and older can make decisions regarding their own health (including participation in health studies), and thus gave consent themselves to participate in the current study. All parents/guardians received written information about the study in advance, as they have the right to be informed.

## Results

### Sample Characteristics

Among the youth@hordaland participants with information about family income, 8,983 (98.1%) had valid responses on the variables of interest. The study sample had a mean age of 17.4 years (standard deviation 0.8), and the sample included more girls (52.9%) than boys (*p* < 0.001). A total of 4.2% reported daily smoking, and 13.7% reported daily snus use. Regarding alcohol, 28.6% reported never having tried alcohol, while 70.9% reported any usual alcohol consumption, and 19.2% reported frequent intoxication. Furthermore, 9.8% reported having tried any drugs, and 20.3% were CRAFFT-positive (summed score of ≥2), indicating potential alcohol- and drug-related problems.

### Classes of Family Income

**FIGURE 1:**
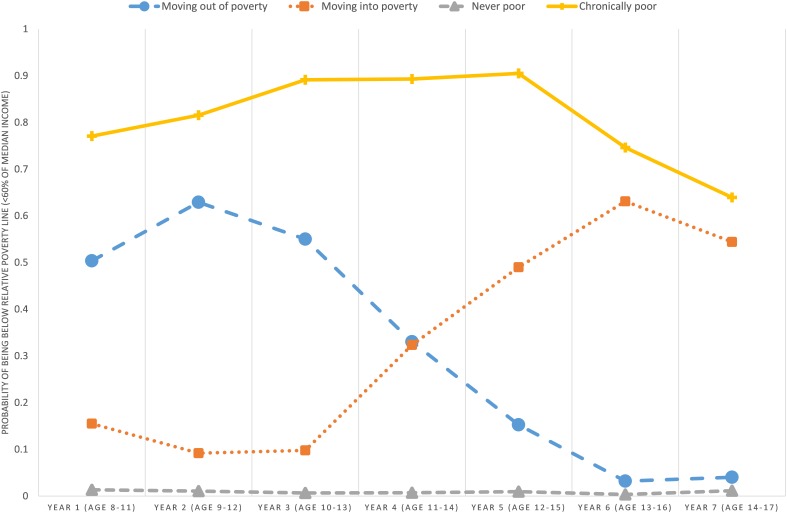
Latent classes across seven time-points from 2004 to 2010 (*N* = 9,154). Groups: ‘moving out of poverty’ (4.6%), ‘moving into poverty’ (3.0%), ‘never poor’ (89.3%), ‘chronically poor’ (3.1%).

Based on an overall consideration of parsimony, fit statistics and meaningfulness, we chose to retain a four-class model (Figure 1). A ‘never poor’ (89.3%) group, followed by two groups characterized by moving in (3.0%) or out (4.6%) of poverty, and a final ‘chronically poor’ (3.1%) group were identified. Neither age nor gender influenced the identification of the classes. The identified classes were found to be comparable across outcomes, and the exclusion of ethnicity in the LCA-estimation did not change the patterning of the classes in any substantial manner.

### Family Income and Subsequent Substance Use

**FIGURE 2:**
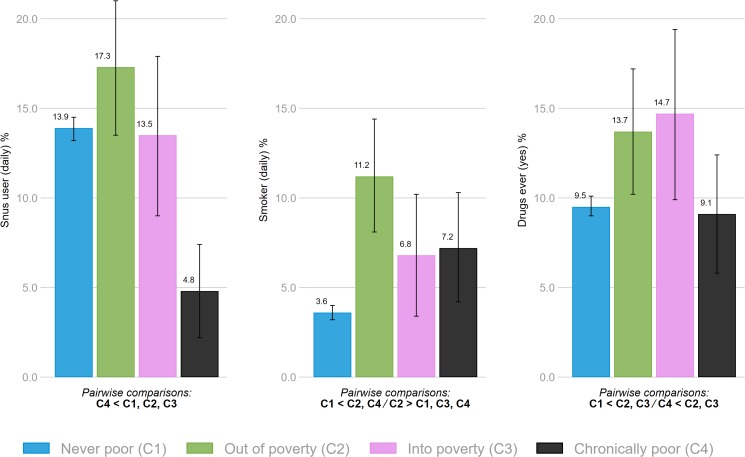
Association between family economic circumstances and snus use, smoking and having ever tried drugs. Bars denote 95% confidence intervals. All indicated pairwise comparisons are significant at *p* < 0.05.

The chronically poor group was *less* likely to report daily snus use, while no significant differences were found between the three other groups (Figure 2). The moving out of poverty-group was *more* likely to report daily smoking compared to all other groups, while the never poor-group were also *less* likely to report daily smoking compared to the chronically-poor group (Figure 2). The proportion having tried drugs were *lowest* in the never poor- and the chronically poor-group, and *highest* among those moving in or out of poverty (Figure 2).

**FIGURE 3:**
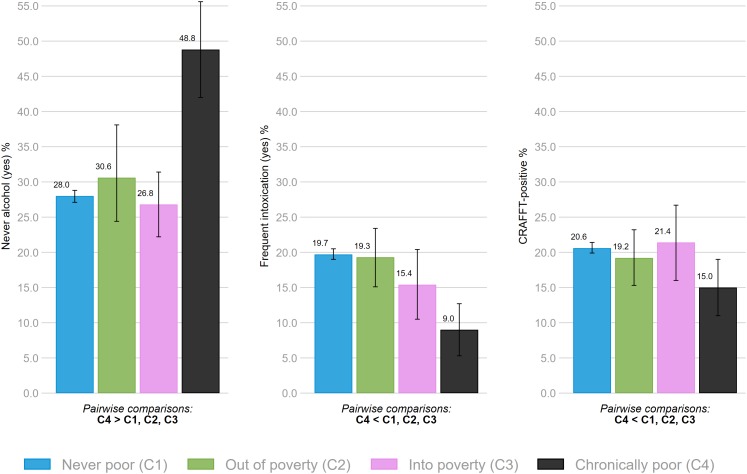
Association between family economic circumstances and having never used alcohol, frequent intoxication and alcohol- or drug problems (CRAFFT-positive). Bars denote 95% confidence intervals. All indicated pairwise comparisons are significant at *p* < 0.05.

With regards to alcohol, the chronically poor group were *more* likely to report never having tried alcohol compared to all other groups (Figure 3). For those with usual alcohol consumption, no statistical differences were found between the groups (data not shown). Reporting frequent intoxication and potential alcohol- or drug problems was *less* likely in the chronically poor-group compared to all other groups (Figure 3). No other differences were identified across the different groups in relation to alcohol use. The test statistics for all associations between family economic circumstances and substance use are summarized in Table 1.

**Table 1:** Association between family economic circumstances and substance use.

Classes	Snus user (% daily)	Smoker (% daily)	Drugs ever (% yes)	Never alcohol (%)	Frequent intoxication (%)	CRAFFT-positive (%)
C1: Never poor	13.9 (13.2–14.5)	3.6 (3.2–4.0)	9.5 (9.0–10.1)	28.0 (27.1–28.8)	19.7 (19.0–20.5)	20.6 (19.9–21.4)
C2: Out of poverty	17.3 (13.5–21.0)	11.2 (8.1–14.4)	13.7 (10.2–17.2)	30.6 (24.4–38.1)	19.3 (15.1–23.4)	19.2 (15.3–23.2)
C3: Into poverty	13.5 (9.0–17.9)	6.8 (3.4–10.2)	14.7 (9.9–19.4)	26.8 (22.2–31.4)	15.4 (10.5–20.4)	21.4 (16.0–26.7)
C4: Chronically poor	4.8 (2.2–7.4)	7.2 (4.2–10.3)	9.1 (5.8–12.4)	48.8 (42.0–55.6)	9.0 (5.3–12.7)	15.0 (11.0–19.0)
Pairwise comparisons	C4 < C1, C2, C3	C1 < C2, C4; C2 > C1, C3, C4	C1 < C2, C3; C4 < C2, C3	C4 > C1, C2, C3	C4 < C1, C2, C3	C4 < C1, C2, C3

## Discussion

### Main Findings

In this large population-based study using self-reported adolescent substance use in linkage to longitudinal registry-based information on family income, we found several differences in substance use across family economic circumstances during childhood. Perhaps not surprisingly, the chronically poor group differed more consistently from the other groups across outcomes. They reported less daily snus use, fewer had tried alcohol, were less likely to report frequent intoxication, and less prone to have potential alcohol- or drug-related problems as indicated by CRAFFT compared to all other groups. They were also less likely to have tried any illicit drug compared to those moving in or out of poverty. Finally, the chronically poor reported more daily smoking than the never poor group, but less daily smoking than the moving out of poverty group. The never poor group was less likely to have tried any illicit drugs compared to the groups moving into or out of poverty, and less likely to smoke daily compared to the moving out of poverty group.

The main findings were similar whether we included ethnicity in the analysis or not, both in terms of classes identified (see Bøe et al., 2017; Sivertsen et al., 2017) and in terms of associations with the substance-related outcomes. There may be several reasons for the lack of impact based on ethnic background. First, most of the participants were designated as Norwegian adolescents (96.3%), limiting the potential overall impact on the analysis. Second, a previous study investigating the association between ethnicity and substance use in the youth@hordaland-survey, found that the observed differences between ethnic Norwegian and ethnic minority adolescents were robust for adjustments for family SES (Skogen et al., 2018). The findings indicate a limited role of SES in the relationship between ethnic background and substance use in the sample.

### Relation to Previous Research

Our findings share some similarities with typical findings from previous research while expanding the knowledge base further. Recent findings suggest that higher SES is associated with less tobacco use [see for instance (Øverland et al., 2010; Charitonidi et al., 2016; Hoebel et al., 2018)], which is broadly similar to our finding that the never poor group was less likely to engage in daily smoking compared to the moving out of poverty and chronically poor group. Interestingly, the group with the highest proportion of daily smokers were those who during childhood moved away from poverty. Less research has focused on the relationship between SES and snus use, but previous findings have suggested a weak negative association or no association with SES (Grotvedt et al., 2008; Øverland et al., 2010). In contrast, our findings suggest a different pattern, where only those who are chronically poor report less daily snus use compared to the other groups. At least two explanations could shed light on these findings. First, the present study is more recent and collected after a marked increase in the use of snus among adolescents in Norway (Folkehelseinstituttet [Norwegian Institute of Public Health], 2017). The secular trend may have changed the association between SES and snus use along with snus use becoming more normal among adolescents. Second, our finding that the chronically poor reported *less* snus use, may be due to ethnic minorities being over-represented in the latter group compared to the other groups (Bøe et al., 2017), as also previous studies have reported substantial ethnic and cultural differences regarding snus use (Grotvedt et al., 2008; Loukas et al., 2012; Skogen et al., 2018). Regarding illicit drug use, previous findings are mixed, perhaps with the exception of the frequently reported inverse association between SES and cannabis use (Daniel et al., 2009; ter Bogt et al., 2014). Our findings indicate that both the never poor and the chronically poor group are less likely to engage in any drug use compared to the two transitional groups. No differences between the two groups at the extreme ends were present with respect to our measure of ever having tried any illicit drugs. Interpretation of our findings is limited in that because we are only able to investigate debut of illicit drugs and do not discern between different types of illicit drugs.

With regards to alcohol, previous studies have demonstrated a complex and multi-layered relationship between SES and alcohol in adolescence (Luthar, 2003; Kendler et al., 2014; Pape et al., 2017, 2018). We found that the chronic poor group were more likely to report never having tried alcohol compared to all other groups. This is contrary to a recent finding from another Norwegian sample of adolescents using parental education as the main indicator of SES among 17 year olds (Pape et al., 2017), but similar to another study using a Norwegian sample with a focus on neighborhood level SES (Pedersen et al., 2015). One possibility may be geographical variations in the association between alcohol and SES within the same country (Pape et al., 2017), or it may be due to different methodological approaches.

Regarding usual alcohol consumption, we found no differences between the groups, which is similar to what Pape and colleagues reported for adolescents in the same age group (Pape et al., 2017), and in an American sample of young adults (Finch et al., 2013). It is, however, not in line with other recent publications with adolescents and young adults who have reported more alcohol consumption among those with high SES (Pedersen et al., 2015; Charitonidi et al., 2016). The chronically poor were also found to be less likely to report frequent intoxication compared to all other groups, a finding which is contrary to a recent publication from Norway (Pape et al., 2018).

For adult alcohol use, a common finding, although not entirely unequivocal, is a positive association with SES – i.e., less alcohol use among those defined as having lower SES, and more alcohol use among those with higher SES (Makela and Paljarvi, 2008; Grittner et al., 2012; Collins, 2016; Lewer et al., 2016; Katikireddi et al., 2017). Despite this, potential alcohol problems and adverse outcomes related to alcohol, seems to be disjoint from the reported association between alcohol use and SES (Collins, 2016). That means that even though more alcohol use is on average reported among those with higher SES, those with lower SES experience more of the potential negative consequences of alcohol use and misuse. This phenomenon – dubbed ‘the alcohol harm paradox’ (Lewer et al., 2016) – has also been observed among adolescents in Norway (Pedersen et al., 2015). Based on CRAFFT, we found an opposite pattern of potential alcohol- or drug problems, where the chronically poor were less likely than any other SES-group to report alcohol- or drug problems as well as less likely to report frequent alcohol intoxication. This may be a correct representation or due to selection effects – but the observation fits well with that findings that substance use was generally less prevalent (with the exception of daily smoking) compared to the never poor group in the present study. It may be that, given the later debut and the lower rate of alcohol use among the chronically poor, it is too early to expect an increase in alcohol and drug-related problems. It would be of interest to follow these adolescents into early adulthood to see if the “the alcohol harm paradox” may be present later. On the other hand, a recent study of the alcohol harm paradox in an adult population (16+ years) observed that different indicators of SES measures appear to influence whether the alcohol-related harm is observed as a linear function across socioeconomic strata or only associated with the most disadvantaged (Beard et al., 2016). This may also explain why we did not observe the alcohol harm paradox in our sample.

### Limitations

The main aim of this study was to investigate associations between trajectories of income and substance use, and therefore we did not assess factors beyond age, gender and ethnic background that may be consequential to actual trajectories themselves. Changes in parental work affiliation or education levels, and structural changes within the family can all lead to changes in family income (Wagmiller et al., 2006). We had no historic information about such events, therefore we were unable to investigate such factors in relation to the different trajectories (Bøe et al., 2017). Second, how we operationalized low income may be viewed as a limitation. Both absolute and relative measures of income have their limitations, but the use of relative measures, as the one used the current study, can be favorable when used within countries to identify those at risk for social exclusion or poverty (UNICEF Innocenti Research Center, 2012).

Third, non-participation can affect the generalizability of the present findings. The response rate for youth@hordaland was ≈53%, with and over-representation of adolescents in schools. Non-participation in survey research is rising (Morton et al., 2012), and non-response is reported to be associated with lower SES (Galea and Tracy, 2007). Thus, the presented results should be considered a lower-bound estimate of the number of adolescents growing up in poor families and the associations reported may be biased as our sample may be skewed toward better SES and psychological health (Bøe et al., 2017). Also, our focus was on family-level SES as measured by tax-based income over time, and it is possible that other contextual SES factors are more important in relation to alcohol and drug use. A recent study found for instance, that the school adolescents (i.e., school-level SES as indicator) attended were more predictive than neighborhood or family income in predicting health-related behavior, including likelihoods of alcohol intoxication and drug use (Coley et al., 2018). Further research should investigate the impact of different contexts in relation to the association between SES and health-related outcomes in adolescents.

### Potential Importance for Public Health

A better understanding of the relationship between indicators of SES during childhood and substance use during adolescence is important in the endeavor to identify individuals and groups at risk for poor health. Adolescent substance use is associated with several negative health outcomes, including mental health problems (Skogen et al., 2014; Pedersen et al., 2018), sleep problems (Sivertsen et al., 2015) and potentially impacts functional outcomes including school performance and attainment (Heradstveit et al., 2017), and work life participation (Henkel, 2011). For instance, a recent study using six samples from Nordic countries reported that use of cigarettes, cannabis, and alcohol was strongly associated with externalizing behavior problems (Pedersen et al., 2018). Furthermore, despite differences between samples in use of substances – as well as national, cultural, and socioeconomic background – very similar associations were reported across samples. Therefore, our detailed and more accurate information of differences in substance use across socioeconomic strata could potentially aid the effectiveness of policy and prevention strategies. This could be done by informing intervention strategies and suggest which groups to target in relation to different aspects and types of substance use. The present findings indicate that there are important differences in substance use and potential problems among adolescents based on family income trajectories during childhood. Previous studies have mainly focused on disadvantaged groups in relation to substance use. Our findings indicate that although this approach may be useful, there is a need for further nuance. The most vulnerable groups in relation to substance use may be the transitional groups in our study. Although the mechanisms behind this observation is not clear, one can speculate that the transition in itself is particularly stressful, or provoke feelings of not belonging.

Poverty’s influence on substance use is not necessarily direct [see for instance (Lee et al., 2018)]. A recent study argued that alcohol problems among adolescents from areas of disadvantage (i.e., areas characterized by low SES) reflects family-based or individual risk-factors, more than neighborhood factors (Pedersen et al., 2015). This suggests that strategies targeting family or individual factors may be a potential avenue in relation to prevention and treatment of alcohol and other drug-related problems. When it comes to identification of individuals at risk for developing alcohol or substance-related problems, multiple SES indicators may be preferable (Patrick et al., 2012). Substance abuse among adolescents has been associated with income at the individual/family-, neighborhood- and school level, and has been observed across the whole economic spectrum, also among those with lower and higher income (Humensky, 2010; Karriker-Jaffe, 2011; Lee et al., 2013; Levine et al., 2018). The important role of peer influences on social norms in in adolescence (West, 1997; Luthar et al., 2013) may suggest that public health interventions targeting harmful substance abuse may best be implemented at the school level.

## Conclusion

Using self-report data on substance use in linkage to longitudinal registry-based family income, we identified important differences in substance use and potential problems among adolescents based on family income trajectories during childhood. Somewhat surprisingly, the chronically poor adolescents had in part lower substance use compared to the other groups. The differential associations between SES and substance use is of public health importance, and they should be examined in further detail in future studies. Specifically, there is a need to gain further knowledge related to how various indicators of SES is associated with substance use, differential associations with different substances, and to which degree age influence the associations.

## Ethics Statement

All participants gave their informed consent upon entering the study. In accordance with the regulations from The Regional Committee for Medical and Health Research Ethics in Western Norway (REC) and Norwegian health authorities, adolescents aged 16 years and older can make decisions regarding their own health (including participation in health studies), and thus gave consent themselves to participate in the current study. Parents/guardians have the right to be informed, and in the current study, all parents/guardians received written information about the study in advance. The study was approved by the REC.

## Author Contributions

JS carried out the literature review for the introduction and discussion sections, and wrote the first draft of the manuscript, while TB performed the data analyses. BS, MH, OH, and TB has been involved in the preparation and planning of the statistical analyses, and have reviewed and contributed to all parts of the written manuscript.

## Conflict of Interest Statement

The authors declare that the research was conducted in the absence of any commercial or financial relationships that could be construed as a potential conflict of interest.

## Footnotes

1 https://helseforskning.etikkom.no/?_ikbLanguageCode=us
